# “Closing the gap in the wrong direction” migration, health policy, and the exclusion of asylum seekers, refugees and undocumented migrants from healthcare access in South Africa

**DOI:** 10.1186/s12889-025-24751-4

**Published:** 2025-11-10

**Authors:** Rebecca Walker, Jo Vearey

**Affiliations:** 1 African Centre for Migration & Society (ACMS), University of the Witwatersrand, PO Box 76, Wits, Johannesburg, 2050 South Africa; 2 Global Health & Migration Unit, Department of Women’s & Children’s Health, Uppsala University, Box 256, Uppsala, 751 05 Sweden

**Keywords:** South africa, Universal health coverage, Migration, Refugees, Asylum seekers, Health policy, Xenophobia, Health equity, Structural violence, Exclusion

## Abstract

**Background:**

The right to health requires that healthcare systems be available, accessible, acceptable, and of good quality for all, regardless of legal or migration status. While South Africa’s Constitution and international commitments uphold this right, recent policy developments—such as the 2023 National Health Insurance (NHI) Act and the 2024 White Paper on Citizenship, Immigration, and Refugee Protection—mark a shift toward institutionalised exclusion. Discriminatory practices that were once informal are increasingly codified in law, aligning healthcare policy with a broader securitisation agenda and undermining progress toward Universal Health Coverage (UHC).

**Methods:**

This paper draws on a multi-method research design combining a structured policy review of South African, regional and global frameworks, with semi-structured interviews (*n* = 25) conducted with healthcare providers, civil society actors, and government stakeholders between 2022 and 2025. Data was analysed using thematic analysis to explore how formal policy and informal practice interact to produce exclusion.

**Results:**

Findings reveal that legal and policy shifts are systematically narrowing healthcare access for asylum seekers, refugees, and undocumented migrants. Four intersecting themes emerged: (1) legal regression and policy securitisation; (2) policy by practice: informal exclusion and administrative discretion; (3) legal liminality and mental health and; (4) civil society as an unsustainable safety net. Despite constitutional protections, migrants are routinely denied care, misclassified, or subjected to unlawful fees. Mental health needs, in particular, remain severely neglected. The study highlights the limits of rights-based advocacy in a hostile policy environment and calls attention to the need for structural reform.

**Conclusion:**

South Africa is not merely failing to meet its UHC goals—it is actively retreating from them. Exclusionary practices, once informal, are now guiding policy. Ensuring healthcare is truly accessible, acceptable, and sustainable for all requires urgent policy, legal, and institutional reforms to protect healthcare access for all, irrespective of status. Without these actions, the most vulnerable will continue to be excluded from essential services, undermining both constitutional values and global health goals.

**Supplementary Information:**

The online version contains supplementary material available at 10.1186/s12889-025-24751-4.

## Background

Achieving Universal Health Coverage (UHC) is a core component of the Sustainable Development Goals (SDGs) and a constitutional commitment in South Africa. Target 3.8 of the SDGs explicitly calls for access to “quality essential health-care services, and access to safe, effective, quality, and affordable essential medicines and vaccines for all” [1–3]. Section 27 of the South Africa Constitution further enshrines the right to access healthcare for “everyone” – without discrimination based on nationality or legal status [4]. Yet in practice, displaced and non-national individuals accessing public healthcare frequently encounter systemic and institutional barriers that restrict their access to essential health services. These exclusions undermine public health outcomes and South Africa’s constitutional and legal obligations and commitments to international human rights law [5–7].

This paper adopts a rights-based lens to examine how healthcare exclusion is produced and sustained for migrant populations in South Africa. While legal categories such as “refugee” or “asylum seeker” are important for determining eligibility and protection under national and international law, this paper uses the term “migrant” as an inclusive, non-legal descriptor encompassing refugees, asylum seekers, and undocumented individuals.^1^ This usage reflects the fluid, overlapping, and often ambiguous legal realities that many people who cross borders experience [8]. Migrants frequently move between legal categories, hold multiple or lapsed statuses or, fall outside formal classifications altogether [8]. Many arrive with valid documentation but become undocumented due to permit expirations, bureaucratic delays, or legal backlogs, rendering them vulnerable to legal liminality, where they exist in an uncertain, in-between status- neither fully recognised or formally excluded - that leaves them vulnerable to discretionary exclusion and discretionary exclusion [9, 10]. This is particularly the case for migrants from neighbouring African countries who face compounded administrative and political exclusion [9, 11].

Although international migrants comprise only 4% of South Africa’s population, they disproportionately encounter barriers to care [12, 13]. These include unlawful charges, denial of services, discrimination, and xenophobic treatment in public health facilities [14]. Marginalised groups within migrant populations – such as (but not limited to) women, LGBTIQ + individuals, those living with disabilities and, informal workers –are particularly vulnerable to intersecting forms of discrimination at the point of care [15, 16].

While these barriers have been widely documented, there has been less critical attention to how legal and policy frameworks actively codify and legitimise exclusion [16, 17]. This paper addresses that gap by examining the institutional, administrative, and legislative mechanisms through which exclusion is structured and reproduced.

Drawing on 25 key informant interviews with healthcare providers, civil society actors, and policy experts, alongside a systematic review of national health and migration frameworks, this study argues that exclusion is not incidental nor simply the result of poor implementation. Instead, it is structurally embedded—reflecting a deliberate narrowing of entitlement that undermines UHC, entrenches inequality, and signals a broader trend of “closing the gap in the wrong direction” (01 KII) [6, 18].

### Global and regional commitments

At the global level, there is broad consensus on the need for inclusive, equitable, and non-discriminatory health systems. The Global Compact for Migration (GCM), the World Health Organization’s Global Action Plan (GAP) for the Health of Refugees and Migrants, and the International Organization for Migration’s (IOM) health strategy all call for migrant integration into national health systems, with legal protections and access to essential services regardless of status [2, 19–21]. Regionally, frameworks such as the African Union’s Migration Policy Framework for Africa (2018–2030), the Africa Health Strategy (2016–2030), and Agenda 2063 echo these principles. They call for regional coordination and the inclusion of displaced populations in national health systems—particularly in response to communicable diseases such as HIV, TB, and malaria [22–24]. The African Charter on Human and Peoples’ Rights (1981) and the Common African Position (CAP) on the Global Compact for Migration further affirm the right of migrants to adequate and affordable healthcare, social protection, and legal assistance [25, 26]. Globally, a growing consensus affirms the need for inclusive, equitable, and non-discriminatory health systems. Frameworks, such as the Global Compact for Migration (GCM), the World Health organisation’s (WHO) Global Action Plan (GAP) for the Health of Refugees and Migrants, and the International Organisation for Migration (IOM) health strategy all call for migrant integration into national health systems, legal protections, and access to essential services regardless of status.

### South Africa’s commitments and emerging contradictions

Post-apartheid South Africa initially embraced a rights-based approach to health enshrined in legislation such as the Refugees Act (1998), the promotion of Equality and Prevention of Unfair Discrimination Act (2000) and the National Health Act (2003), all of which reflect a commitment to universality and inclusion [17, 27, 28]. South Africa is also signatory to a number of international and regional human rights instruments mandating equitable access to care for all [19, 29, 30].

However, recent legislative and policy developments signal a retreat from these commitments. The 2020 Border Management Authority Act (BMA), the 2023 National Health Insurance (NHI) Act and, the 2024 draft White Paper on Citizenship, Immigration, and Refugee Protection increasingly tie access to healthcare to documentation and legal status–contradicting constitutional guarantees and aligning with a global trend toward securitised migration governance [17, 31, 32]. These shifts mirror international patterns where migration is framed as a threat, and access to services is reframed as privilege rather than a right [14, 33, 34].

### Institutional barriers and xenophobic politics

These exclusions are further compounded by systemic weaknesses in the public health system. As the gap between the wealthy and poor in South Africa increases the chronically underfunded public health sector is required to serve approximately 84% of the population [35]. The well-resourced private sector meanwhile. caters to a small, insured minority [36]. The public sector faces critical infrastructure decay, staff shortages, and medicine stockouts – challenges that have undermined the quality of care across core dimensions such as safety, accessibility, and equity [37, 38]. The recent freeze on U.S. global health funding – particularly for HIV and sexual and reproductive health (SRH) - threatens to exacerbate these weaknesses, placing additional strain on an already overstretched system [39].

In this context of scarcity, migrants are increasingly scapegoated and framed as “undeserving” of care [6, 40, 41]. Popularist and xenophobic movements such as Operation Dudula have normalised anti-migrant sentiments public harassment and inciting violence, social media campaigns, and attacks on clinics - often tacitly endorsed or even instigated by political leaders [42–44]. This rhetoric has permeated institutional culture, shaping frontline behaviour, policy design, and public opinion [7, 45, 46].

### From informal exclusion to legal regression

This study contributes to a growing body of evidence that informal, discretionary exclusion is being codified in law. Practices that were once inconsistent or ad hoc – such as denial of care based on documentation status – are increasingly sanctioned by formal policy. Laws like the NHI Act and the draft 2024 White Paper mark a decisive shift: from healthcare as a universal right to a conditional, securitised entitlement. This process exemplifies what scholars term “legal regression,” wherein progressive constitutional norms are gradually eroded through legal and administrative reform [16, 47, 48].

As the findings will show, this is not simply a case of implementation failure, but a structural and strategic reconfiguration of healthcare governance. Rather than narrowing the gap between law and practice, South Africa is “*closing the gap in the wrong direction*”—undermining universality and redefining healthcare access in increasingly exclusionary terms. This paper explores the implications of this shift, arguing that unless actively reversed, it risks setting a dangerous precedent in which access to care is contingent not on health need, but on legal status and political expedience.

## Methodology

### Study design

The findings in this paper are drawn from research conducted between 2020 and 2025, undertaken in two phases. The initial phase (2020–2023), involving the authors and non-author contributors (listed in the Acknowledgement section), was conducted as part of the GCRF Protracted Displacement project, ‘Improving healthcare at the intersection of gender and protracted displacement amongst Somali and Congolese refugees and Internally Displaced Persons (IDPs) (DiSoCo)’.^2^ The second phase (2023–2025), including an updated review of global and regional governance frameworks and an updated review for South Africa, was undertaken by the authors as part of the Gendered violence and poor mental health among migrants in precarious situations Global Health Research Group (GEMMS).^3^ This study employed a qualitative design informed by a rights-based and critical policy analysis framework.

### Data collection

Two primary data sources were used: Key Informant Interviews (KIIs) and a policy and legislative review.

#### Key informant interviews

A total of 25 semi-structured interviews were conducted with stakeholders involved in health service care delivery, policy development, and advocacy. Participants included public sector healthcare providers, civil society representatives, legal experts, frontline workers, and policy advisors. Questions concerned issues such as healthcare provision generally and mental healthcare specifically for displaced populations in South Africa (Congolese and Somali), South Africa’s healthcare system in policy and practice, the connections and disconnections between the healthcare system and mental health provision^4^. A purposive sampling strategy was used to ensure representation across sectors and regions, with additional snowball sampling to reach informants with specialised knowledge. Interviews were audio-recorded with consent. Ethical approval was obtained from the University of the Witwatersrand and participants were anonymised using non-identifiable codes.^5^

#### Policy and legislative review

A structured review was conducted of national laws, policy documents, regulations, strategic frameworks, and ministerial directives relevant to health, migration, and human rights. This included the Constitution (1996), the National Health Act (2003), Refugees Act (1998), Immigration Act (2002), the National Health Insurance Act (2023), and the draft White Paper on Citizenship, Immigration and Refugee Protection (2024), among others (see Appendix A). Regional and international instruments such as the SDGs, the Global Compact for Migration, the African Union’s Migration Policy Framework and various Southern African Development Community (SADC) policies and frameworks were also examined for comparative and normative reference.

### Data analysis

Interviews were audio-recorded with consent, transcribed verbatim, and coded using reflexive thematic analysis, following the six-phase approach outlined in Braun and Clarke [49, 50]. Analysis was iterative and interpretive, involving repeated reading, open coding and thematic development. Codes were initially generated inductively from the data, and cross-validated against policy documents to identify points of alignment, contradiction, or omission. Attention was paid to structural drivers of exclusion, including securitisation, legal liminality, and interdepartmental incoherence. The analysis aimed to reflect both institutional and frontline perspectives while remaining grounded in the lived realities described by participants.

**Fig. 1:**
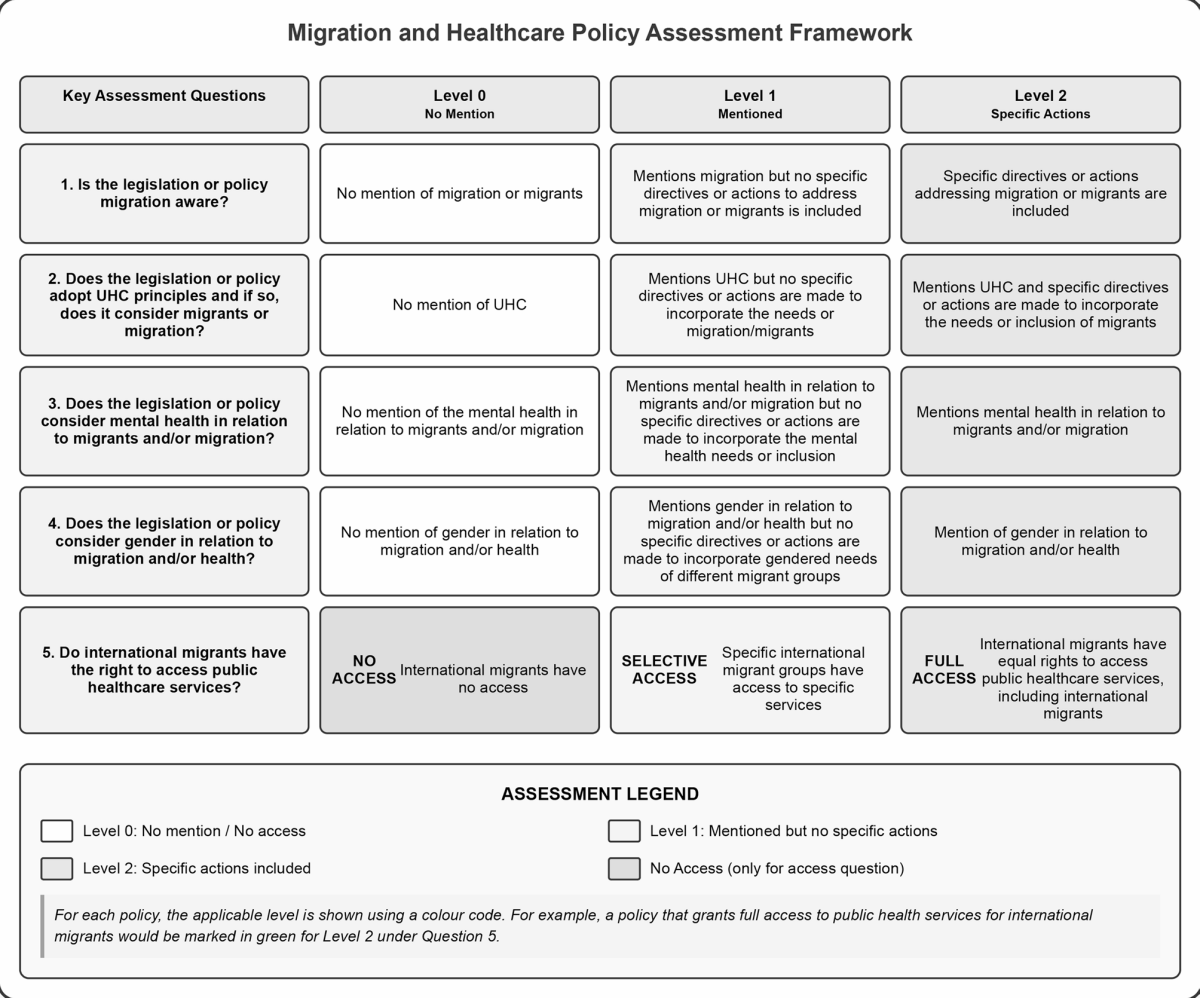
Assessment framework^6^

Global and regional instruments were identified and reviewed iteratively, with legal and policy texts systematically assessed for alignment with South Africa’s constitutional obligations and international commitments, including UHC targets and core human rights principles. The findings were synthesised using a structured assessment framework (see Fig. 1), which enabled comparative analysis across key governance areas. These included: [1] recognition and protection of the right to health for migrants and displaced populations; [2] integration of migration into healthcare planning and access to public health services; [3] inclusion of mental health and psychosocial support within broader health system strategies; [4] adoption of gender-sensitive approaches, including protections for displaced women, girls, and gender-diverse groups.

### Reflexivity and limitations

While the research captures a broad institutional landscape, access to senior government officials—especially those involved in high-level policy design and implementation—was limited. This limitation reflects broader trends of institutional defensiveness around migration governance and should be acknowledged as a constraint on the data. Additionally, while the study focuses on systemic and institutional dimensions of exclusion, it does not directly include the voices of migrant individuals themselves – these can be found in other papers by the authors [51].

The fieldwork was also impacted by the context of the COVID-19 pandemic and the strict “lockdown” imposed by the Government to prevent the spread of the virus from March -May 2020, which meant no one could leave their homes and all businesses, schools and public services were closed. Key informant interviews had to be moved online and a number of the potential stakeholders, especially those working in the health system, were unavailable or – minimally available. While this posed limitations to the number of interviews and range of participants it also presented important insights on pandemic preparedness and migration governance in a time of crisis [52, 53]. While this paper does not focus explicitly on COVID-19, the broader impacts of the pandemic on the health system and migrant groups are recognised and have been explored in complementary studies [34, 51, 54].

The lead researcher approached the study from a critical, rights-based perspective, shaped by prior engagement with civil society organisations (CSOs) working on migration and health. Given the researcher’s ongoing work in this field and established relationships with many civil society actors, positionality was carefully considered and reflected upon throughout data collection and analysis. Attention was paid to how these relationships might influence access, interpretation, and representation of findings.

The analysis also took into account participants’ differing proximities to institutional power, recognising how these positional dynamics shaped their perspectives. This was considered alongside the significant pressures faced by both civil society actors and healthcare workers operating in a context of growing need, shrinking resources, and reduced institutional support.

## Results

Analysis of 25 key informant interviews and national policy documents revealed four interrelated themes that explain how healthcare exclusion for cross-border migrants in South Africa is produced, sustained, and increasingly codified. These themes are:

1. Legal regression and policy securitisation;
2. Policy by practice: informal exclusion and administrative discretion;
3. Legal liminality and the psychosocial cost of exclusion; and.
4. Civil society as an unsustainable safety net.

### Legal regression and policy securitisation

**Fig. 2:**
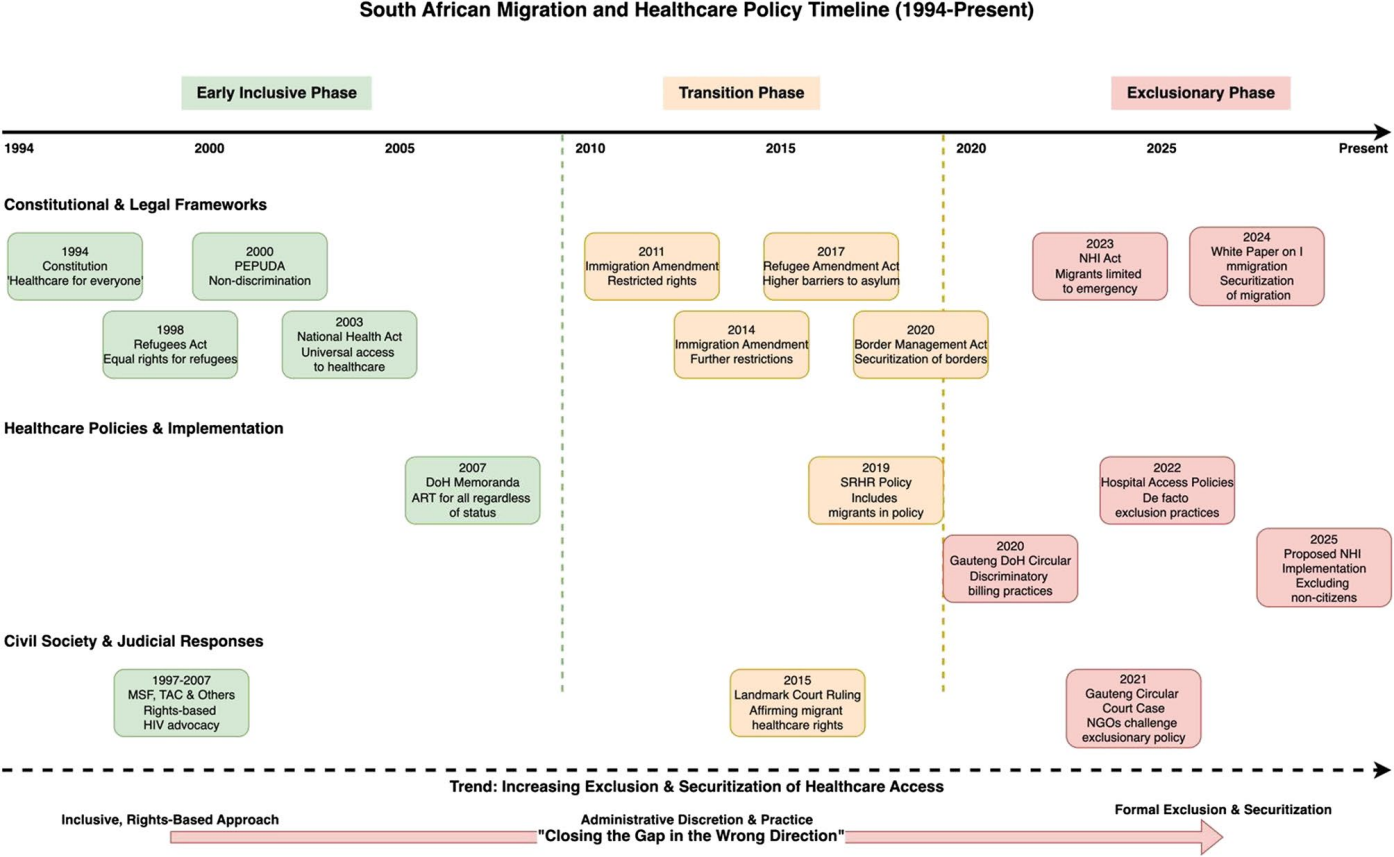
South African migration and health policy timeline (1994- present: select policies)

Participants consistently described a shift from rights-based health governance to a securitised policy regime in which migration is increasingly framed as a threat. Recent legislation—particularly the 2023 National Health Insurance (NHI) Act and the draft 2024 White Paper on Citizenship, Immigration, and Refugee Protection—was seen to formalise exclusionary practices that previously occurred informally [17, 32, 55]. These laws explicitly tie access to healthcare to legal status, contradicting constitutional guarantees of universality and undermining international obligations. As illustrated in Fig. 2, this shift marks a broader legal regression.

While this trend is dominant, exceptions remain. The draft National Labour Migration Policy (NLMP) and the 2019 National Integrated Sexual and Reproductive Health and Rights (SRHR) Policy retain rights-based commitments. The SRHR Policy explicitly names migrants and asylum seekers as a priority group and outlines culturally competent, gender-sensitive service provision, while the NLMP affirms equal treatment and healthcare access for migrant workers [56, 57].

This policy tension—between progressive legal precedent and exclusionary legislative reform—was reflected in participants’ accounts. One health stakeholder recalled:

“They [the DoH] know and recognise the importance of addressing migrant health, especially on HIV and other communicable diseases… Hard lessons were learned in those terrible HIV years, and we built relationships and worked collaboratively.” (KII 04).

Yet the same respondent noted a dramatic policy reversal:

“We’ve seen this willingness, this clear engagement and oversight fall away… people in powerful government positions now choose to ignore those lessons, and we focus instead on excluding people as an answer to deep systemic issues.” (KII 04).

Others voiced frustration with the state’s disengagement from public consultation. One participant noted: “Everyone—and their mother—sent in submissions on the draft NHI Bill… but this didn’t result in any substantive changes.” (KII 02).

The NHI Act was described as “inhumane” (KII 02) and “a huge concern in terms of rolling back rights” (KII 03). Similarly, the 2020 Border Management Act (BMA) and the draft 2024 White Paper were seen to reflect a broader securitisation agenda: “They [government] are using narratives like trafficking—sometimes with inflated figures—to justify militarising the borders.” (KII 01).

Together, these developments reflect a structural shift in which migrants are redefined as threats rather than rights-holders, reshaping the foundational principles of South Africa’s healthcare system.

### Policy by practice: informal exclusion and administrative discretion

Even when formal protections exist, frontline implementation frequently diverges from the law. Respondents described how healthcare workers act as gatekeepers - denying care based on personal bias, misinformation, or pressure from institutional culture:

“On the ground, it doesn’t really matter what’s written on paper… people just make decisions based on what they want to be doing” (02 KII).

The 2020 Gauteng Department of Health (DOH) circular, which limited care to citizens, and documented migrants, was repeatedly cited [58]. Although later overturned in court, its informal enforcement persists. As respondents explained: “The policy has done its damage… we know it is still being implemented, even to this day” (05 KII) and “Even policy circulars that may be later withdrawn can have a lasting damaging impact” (02 KII).

Documentation emerged as a critical barrier. During COVID-19, many migrants with official permits were charged full private fees in public hospitals despite legal extensions^7^: “We are now fighting these battles with CEOs… despite the extension… they say they received explicit instruction from the DoH to charge them” (06 KII).

The consequences are often severe. One case involved a migrant woman denied a birth certificate for her new-born due to an unpaid hospital bill exceeding R120,000 – highlighting the legal and social implications of administrative exclusions. Respondents saw this not merely as bureaucratic failure, but as a systemic effort to deter migrants from seeking care: “It’s a calculated and deliberate attempt to exclude migrants from care” (04 KII).

### Legal liminality and mental health: the psychological toll of exclusion

Mental health emerged as a particularly acute site of exclusion. Although the Mental Health Care Act (2002) promotes community-based care, respondents highlighted limited implementation and severe underfunding, especially in migrant communities: “A big challenge is the capacity of district and provincial health managers to actually implement and plan mental health services that are integrated into primary care.” (08 KII). Another respondent noted: “You will look for mental health services in all the provinces, and the problem is there – you won’t find them - it is a struggle in the system” (01 KII).

Migrants were viewed as almost entirely absent from policy frameworks: “Thinking about the Mental Health Policy… there’s very little attention paid to migrants” (07 KII).

Mental health services were widely described as under-resourced and difficult to access, even for South African citizens. Meanwhile, referrals systems were described as largely ineffective: “We must have referred, let me say between fifty and a hundred people into the clinic system… only five or six ever got an appointment” (08 KII).

Stigma compounds these gaps. One respondent recalled a migrant man seeking support for depression being told: “If you want to kill yourself, just kill yourself.” (08 KII).

Legal liminality—particularly around documentation—emerged as a major driver of chronic stress and declining mental health- captured in the following comment: “The process of obtaining or renewing documentation is a trauma in of itself” (08 KII).

Ultimately respondents described the mental health system as collapsing under the weight of unmet need: “Whether you’re a South African or not—the whole GBV scene, the mental health scene in South Africa—is just a disaster” (09 KII).

### Civil society as an unsustainable safety net

Respondents highlighted the essential role of civil society in filling service gaps left by the state. NGOs provide care, advocate for accountability, and serve on policy platforms: “We sit on various Government platforms…we’ve worked very closely with DOH and the partnership has been quite constructive” (02 KII).

Yet these efforts are often temporary and under-resourced as one respondent noted: “We knew we weren’t going to be able to like really set up a structured project that could make significant changes…this was a stop-gap”(02 KII).

The relationship between civil society and the state was therefore described as one of triage rather than meaningful reform: “You’re relying on organisations that are overworked, burned out, and underfunded” (16 KII) “we’re constantly plugging holes…it’s not sustainable” (03 KII).

Legal strategies were also seen as limited: “You can litigate and litigate but if they [the government] chose not to listen… in fact chose to deliberately defy the courts, then what can you do?” (01 KII).

Some respondents argued for shifting advocacy strategies away from solely rights-based framings:

“This whole idea of rights… unless you actually work with the ugly shit of the violence and the xenophobia… I don’t think legislation and banging on about rights is actually going to shift people” (12 KII).

Others called for a pragmatic, intertest-based appeals: “We need to start talking in terms of cost-effectiveness, not just rights.” (17 KII).

## Discussion

This study demonstrates how South Africa’s healthcare system is undergoing a troubling reversal. Rather than closing gaps in health access, recent policy and practice reforms are entrenching exclusion—especially for asylum seekers, refugees, and undocumented migrants. These findings underscore a structural transformation in which universality, once embedded in post-apartheid health governance, is being replaced by conditional, status-based access. In this sense, the country is not simply failing to meet its Universal Health Coverage (UHC) commitments—it is, as one respondent put it, “closing the gap – but in the wrong direction” (01 KII).

### Securitisation, discretion, and the reconfiguration of universality

South Africa reflects a growing global trend in which healthcare systems are increasingly shaped by the securitisation of migration governance. As Scheel and Squire argue in their theory of the “diffused border,” migration control is no longer confined to territorial frontiers—it is dispersed across public institutions, including health systems [59]. The NHI Act (2023) and the draft White Paper on Citizenship, Immigration and Refugee Protection (2024) exemplify this institutionalised securitisation, reconfiguring access to healthcare around legal status and embedding exclusion within the infrastructure of care.

This mirrors global shifts where migrants are portrayed as threats to national welfare systems, and access to public services becomes contingent on immigration status [47, 60]. In this securitised context, health systems increasingly function as mechanisms of internal border control, rather than inclusive platforms for public health delivery [61]. These dynamics have been widely documented in high-income countries such as the UK and the USA—but are also evident in low- and middle-income countries, including South Africa, where rising populism and administrative fragmentation enable exclusionary reforms [61–63].

In South Africa, this formal codification of exclusion now intersects with entrenched patterns of everyday administrative discretion. Even where legal rights to healthcare are guaranteed—through the Constitution, the Refugees Act, or court judgments—real access often depends on localised decision-making [16, 64]. These practices exemplify what Lipsky famously termed “street-level bureaucracy,” and what more recent migration scholars describe as “policy by practice”: a governance mode in which informal norms, discretionary enforcement, and operational interpretation determine who gets access to services—regardless of what the law stipulates [65]. However, this study shows that South Africa is not only witnessing informal erosion of rights; it is also experiencing a convergence between exclusionary practice and exclusionary law. Migrants and asylum seekers are increasingly excluded not through gaps in enforcement but through deliberate legislative design.

This codification of exclusion - signals what legal scholars describe as “legal regression” or the “unmaking of rights” [16, 47]. Rights are not merely undermined through poor implementation, but actively redefined through legal mechanisms – such as the NHI - that directly contradict their universality. For South Africa especially, this formal retreat from rights-based governance is particularly significant given the country’s post-apartheid history and leadership position within SADC on human rights [6, 41].

### Mental health as an indicator of structural violence

Mental health emerged as one of the most neglected dimensions of health service delivery for displaced populations. Despite policy commitments to integrating mental health into primary healthcare, migrants are rendered effectively invisible—not only in mental health strategies, but also in the design of community-based care and psychosocial support mechanisms [66, 67]. In the South African context, this dynamic is amplified by an already fragile and under-resourced public mental health sector [68, 69].

The psychological toll of displacement—marked by chronic stress, anxiety, and trauma linked to legal liminality—is compounded by systemic neglect and stigma at the point of care as described in the results. Legal liminality refers to a state of prolonged uncertainty in which individuals occupy an ambiguous position between legality and illegality. For migrants, this leads to significant precarity, restricting access to rights and protections while heightening vulnerability to exclusion [10, 70]. In South Africa, persistent dysfunction within the immigration system, frequent changes to regulations, and restrictive amendments to the Refugee Act have entrenched this condition, creating cycles in which certain migrants move in and out of formal status. As highlighted in the policy review, this “in-betweenness” is not always temporary but can be deliberately maintained by states as a governance strategy compounding both material insecurity and mental distress [71]. These findings align with international evidence showing that migrants’ mental health deteriorates when structural violence intersects with exclusionary service systems [48, 72–75].

This exclusion is emblematic of structural violence—the slow, often invisible erosion of dignity, wellbeing, and survival through policies and institutional inaction [54, 76, 77]. The failure to account for the mental health needs of displaced people thus constitutes not only a policy oversight but a profound human rights concern. While globally, the WHO and the IOM have called for mental health strategies that explicitly respond to the trauma of displacement, detention, and legal uncertainty in the absence of adequate investment and deliberate inclusion, such frameworks remain aspirational [78–80].

### Strategic pathways: beyond rights

While South Africa’s constitutional framework provides a strong legal foundation for the right to health, this study highlights the limitations of legal guarantees in the absence of political will, institutional accountability, and operational coherence. More critically, it underscores a growing discursive shift: migrants are increasingly viewed not as rights-holders, but as burdens to be managed or excluded [6]. In this context, achieving meaningful inclusion for displaced populations requires a pragmatic shift—from reliance on rights-based discourse alone to strategies that also appeal to public health imperatives, economic rationality, and broader societal risk [47].

#### Interdepartmental coordination

Several strategic pathways emerge. First, interdepartmental coordination—especially between the DoH and the DHA—must be urgently improved to address legal liminality, harmonise documentation procedures, and clarify eligibility in ways that are consistent across institutions.

#### Realign reforms with constitutional obligations

Second, national reforms such as the NHI must be re-aligned with South Africa’s constitutional and international obligations. The current trend toward securitised and exclusionary migration-health policy contradicts the very principles underpinning UHC. Unless corrected, such reforms will reinforce inequality and institutionalise fragmentation [55, 81].

#### Engage regional institutions

Third, regional institutions—including the African Union, SADC, and IGAD—must move from norm-setting to enforcement. While continental frameworks on health and mobility are robust in principle, they require concrete mechanisms for monitoring, benchmarking, and holding states accountable to their commitments [33].

Civil society continues to play an indispensable role in filling service gaps, providing advocacy, and challenging policy regressions. However, as political hostility increases and donor funding contracts, this model is no longer sustainable. Reliance on overburdened civil society actors risks masking systemic failure and reinforcing the perception that migrant healthcare is an exceptional rather than routine responsibility. Similar dynamics have been documented in other low- and middle-income countries, where non-state actors serve as “shock absorbers” within fragile health systems [7, 82, 83].

Respondents also noted a strategic pivot in advocacy—from rights-based claims to arguments based on public health and cost-effectiveness. While this pragmatism may enhance policy traction, it reflects a deeper crisis: when governments selectively ignore constitutional rulings and rights-based obligations, the legitimacy of the legal order itself is called into question [41, 55].

## Conclusion: “closing the gap in the right way”

South Africa’s health policy trajectory reveals a fundamental contradiction: while constitutional and global commitments espouse universality, recent legislative reforms institutionalise exclusion. This paper has shown that the denial of healthcare to asylum seekers, refugees, and undocumented migrants is no longer incidental—it is becoming a central feature of national policy. The NHI Act (2023) and the draft White Paper (2024) mark a shift from implicit to explicit exclusion, aligning healthcare governance with a securitised approach that treats legal status as the gateway to entitlements.

These reforms reflect and reinforce broader global trends, yet they are particularly troubling in South Africa’s post-apartheid context, where the Constitution promises non-discrimination and access to care for all. The findings demonstrate how informal, frontline exclusions—shaped by administrative discretion, institutional ambiguity, and xenophobia—now converge with legal reforms to produce what can be termed “structured exclusion.” The erosion of migrant mental health, alongside the systemic invisibility of displaced populations in service planning, illustrates the human cost of this policy shift.

Despite the efforts of civil society, exclusion is deepening. Without urgent action to reverse this trajectory, UHC will remain out of reach—offered in rhetoric but denied in practice. South Africa must reassert the principle that healthcare is a right, not a privilege. This means integrating migrant needs into national health planning, improving interdepartmental coordination, ensuring accountability for rights violations, and resisting securitised governance logics. Only then can the gap between law and practice begin to close—in the right direction.

## Supplementary Information

Supplementary Material 1.

Supplementary Material 2.

## Data Availability

The following supporting information can be downloaded at: https://shorturl.at/v9BPR, Figure 1: Assessment Framework; Figure 2: South African Migration and Health Policy Timeline 1994- Present - select policies. Table 1: Table 1: Key Laws and Policies on Health Access and Migration in South Africa.

## Appendix A

**Table 1:** Key laws and policies on health access and migration in South Africa

Law/Policy	Date	Key inclusive/restrictive measures for migrants to access healthcare in South Africa
The Constitution (Sect. 27)	1994	Guarantees the right to healthcare for all, regardless of legal status.
The Immigration Act (2002, amended 2004)	2002	Access to healthcare is determined by legal status. State institutions are required to report “illegal foreigners” or individuals with unclear status to the Director-General.
The National Health Act	2003	Recognises the health needs of vulnerable groups. Provides free healthcare for pregnant women and children under six.
The Refugee Act	1998	Grants refugees the same legal entitlements as South Africans (excluding political rights).
Promotion of Equality and Prevention of Unfair Discrimination Act	2000	Prohibits denial of healthcare access on listed grounds (e.g., sex, social origin).
NDoH Memoranda (2006) and Directives (2007)	2006/2007	States that migrants without South African IDs should not be denied antiretroviral treatment (ART) in public health facilities.
Immigration Amendment Acts (2007, 2011) & Immigration Regulations (2014)	2007–2014	Ensures detainees have access to basic health services. Requires separation based on health and security risk categories.
Refugee Amendment Act (2017) & Regulations (2019)	2019	Narrows asylum seeker protections, including limits on healthcare, education, and employment rights.
National Integrated Sexual and Reproductive Health and Rights Policy	2019	Recognises migrants and asylum seekers as a priority group. Promotes culturally competent, migrant-friendly services and continuity of care.
Border Management Act	2020	Increases securitisation; restricts mobility and access to services for non-citizens.
National Strategic Plan for the Prevention and Control of Non-Communicable Diseases (2020–2025)	2020	Outlines goals for achieving SDGs but makes no specific provision for non-citizens.
Labour Migration Policy (Draft)	2022	Proposes pathways to Universal Health Coverage (UHC) and SDG targets, with a focus on migrant labour inclusion.
National Health Insurance (NHI) Act	2023	Restricts healthcare access for non-citizens to emergency and notifiable conditions only.
White Paper on Citizenship, Immigration, and Refugee Protection (Draft)	2024	Proposes stricter immigration criteria. Reviews South Africa’s international refugee commitments to align with national capacity.

The table below identifies the key laws and policies governing health access and migration in South Africa and the inclusive or restrictive measures in terms of migrants access to public healthcare

## Abbreviations

AU: African Union
BMA: Border Management Authority
CSO: Civil Society Organisation
DHA: Department of Home Affairs
GEMMS: Global Health Research Group
ICESC: International Covenant on Economic, Social and Cultural Rights
IDP: Internally Displaced Persons
KII: Key Informant Interview
LGBTIQ+: Lesbian Gay Bisexual Transgender and Queer
NLMP: National Labour Migration Plan
SRHR: Sexual Reproductive and Health Rights
UHC: Universal Health Coverage

## Footnotes

1 A refugee is defined as an individual recognised as having a well-founded fear of persecution based on race, religion, nationality, membership in a particular social group, or political opinion, and who is unable or unwilling to return to their home country. An asylum seeker is a person who has left their country of origin and is seeking international protection but whose claim for refugee status has not yet been determined. An undocumented person is an individual who is present within a state’s territory without lawful immigration status as defined by national legislation. This includes persons who entered without inspection, remained after the expiration of a visa or permit, or whose applications for asylum, residency, or other legal status have been denied or withdrawn.

2 The research was supported by a grant (reference number *ES/T004479/1*) from the UK Economic and Social Research Council (ESRC) via the Global Challenges Research Fund (GCRF) Development-based approaches to Protracted Displacement scheme.

3 Supported by the NIHR (grant ref: NIHR134629).

4 See interview guide in Supplementary file 1.

5 Protocol code HRECNM20-09-048, September 2020.

6 Figure 1 presents the categories (levels) used to analyse the laws and policies, with five assessment questions listed in the rows. The categories indicate ratings from Level 0—where a policy does not mention the issue—to Level 2—where the issue is fully addressed through specific directives

7 During the pandemic, the governments enforced strict lockdown measures to prevent further spread of the virus. With all business, public institutions and public spaces closed migrants needing to renew their permits were unable to access the Refugee Reception Offices (RRO) and Department of Home Affairs.
