# Supplementary material for: “Closing the gap in the wrong direction” migration, health policy, and the exclusion of asylum seekers, refugees and undocumented migrants from healthcare access in South Africa
Source: BMC Public Health. 2025 Nov 10;25:3877. doi: 10.1186/s12889-025-24751-4 (PMC12604325; doi:10.1186/s12889-025-24751-4)

Figure 1: Assessment Framework

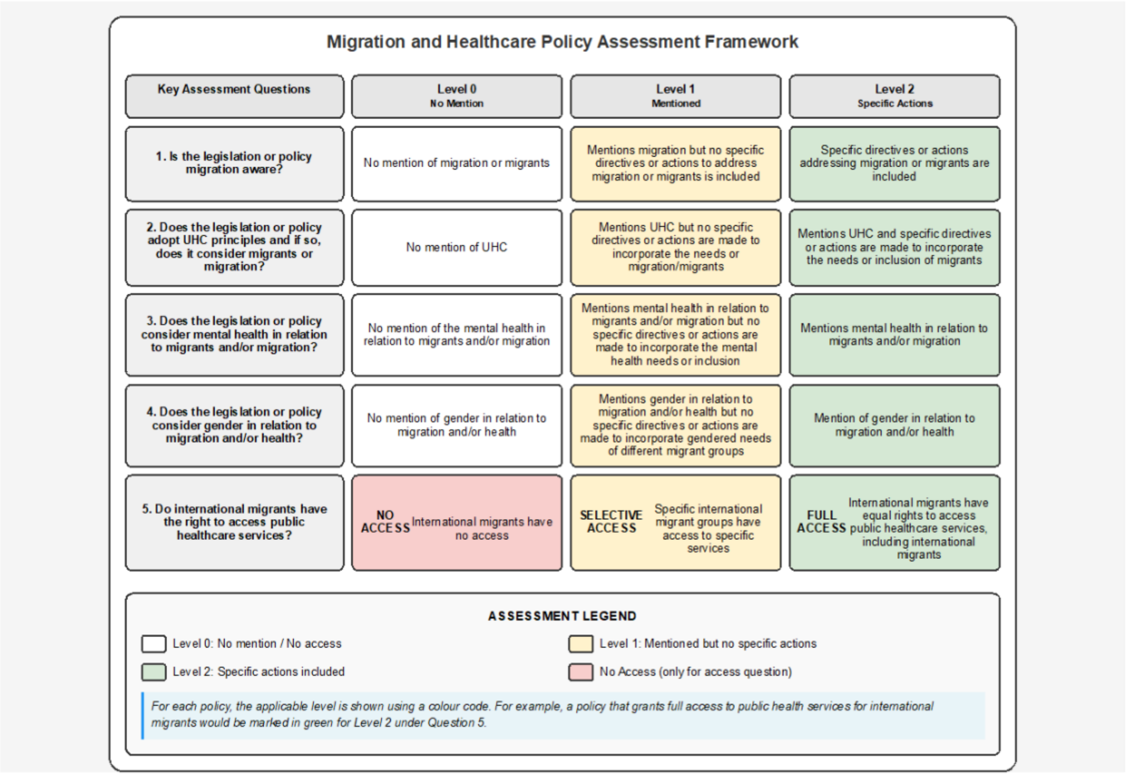

Migration and Healthcare Policy Assessment Framework

Key Assessment Questions	Level 0 No Mention	Level 1 Mentioned	Level 2 Specific Actions
1. Is the legislation or policy migration aware?	No mention of migration or migrants	Mentions migration but no specific directives or actions to address migration or migrants is included	Specific directives or actions addressing migration or migrants are included
2. Does the legislation or policy adopt UHC principles and if so, does it consider migrants or migration?	No mention of UHC	Mentions UHC but no specific directives or actions are made to incorporate the needs or migration/migrants	Mentions UHC and specific directives or actions are made to incorporate the needs or inclusion of migrants
3. Does the legislation or policy consider mental health in relation to migrants and/or migration?	No mention of the mental health in relation to migrants and/or migration	Mentions mental health in relation to migrants and/or migration but no specific directives or actions are made to incorporate the mental health needs or inclusion	Mentions mental health in relation to migrants and/or migration
4. Does the legislation or policy consider gender in relation to migration and/or health?	No mention of gender in relation to migration and/or health	Mentions gender in relation to migration and/or health but no specific directives or actions are made to incorporate gendered needs of different migrant groups	Mention of gender in relation to migration and/or health
5. Do international migrants have the right to access public healthcare services?	NO ACCESS International migrants have no access	SELECTIVE ACCESS Specific international migrant groups have access to specific services	FULL ACCESS International migrants have equal rights to access public healthcare services, including international migrants

ASSESSMENT LEGEND

- Level 0: No mention / No access
- Level 1: Mentioned but no specific actions
- Level 2: Specific actions included
- No Access (only for access question)

For each policy, the applicable level is shown using a colour code. For example, a policy that grants full access to public health services for international migrants would be marked in green for Level 2 under Question 5.

Figure 2: South African Migration and Health Policy Timeline (1994- Present - select policies)

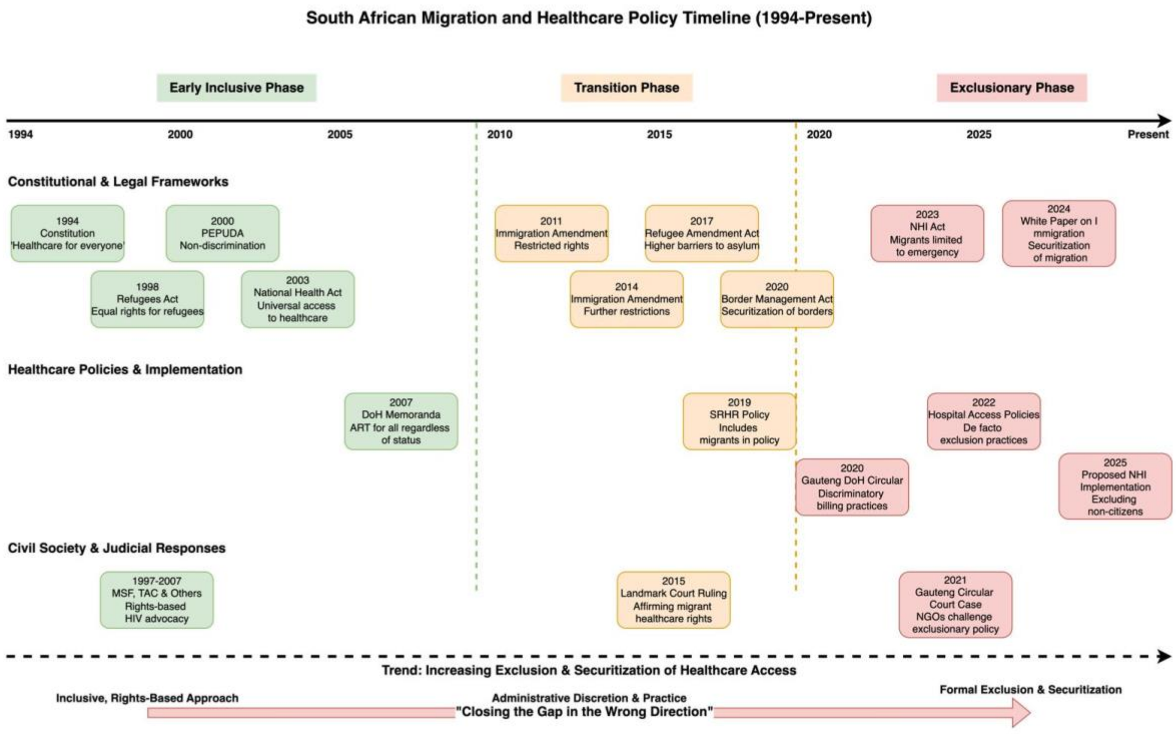

Supplement: Supplementary file 1 — Supplementary Material 1. [file 12889_2025_24751_MOESM1_ESM.zip › Figures 1 and 2 Table 1 /Figure 1 and Figure 2.pdf]
