# Supplementary material for: “Closing the gap in the wrong direction” migration, health policy, and the exclusion of asylum seekers, refugees and undocumented migrants from healthcare access in South Africa
Source: BMC Public Health. 2025 Nov 10;25:3877. doi: 10.1186/s12889-025-24751-4 (PMC12604325; doi:10.1186/s12889-025-24751-4)
Supplement: Supplementary file 1 — Supplementary Material 1. [file 12889_2025_24751_MOESM1_ESM.zip › Figures 1 and 2 Table 1 /Table 1- Key Laws and Policies on Health Access and Migration in South Africa.pdf]

## Table 1: Key Laws and Policies on Health Access and Migration in South Africa

| Law/Policy | Date | Inclusions / Restrictions |
|---|---|---|
| The Constitution (Section 27) | 1994 | Guarantees the right to healthcare for all, regardless of legal status. |
| The Immigration Act (2002, amended 2004) | 2002 | Access to healthcare is determined by legal status. State institutions are required to report “illegal foreigners” or individuals with unclear status to the Director-General. |
| The National Health Act | 2003 | Recognizes the health needs of vulnerable groups. Provides free healthcare for pregnant women and children under six. |
| The Refugee Act | 1998 | Grants refugees the same legal entitlements as South Africans (excluding political rights). |
| Promotion of Equality and Prevention of Unfair Discrimination Act | 2000 | Prohibits denial of healthcare access on listed grounds (e.g., sex, social origin). |
| NDoH Memoranda (2006) and Directives (2007) | 2006 / 2007 | States that migrants without South African IDs should not be denied antiretroviral treatment (ART) in public health facilities. |
| Immigration Amendment Acts (2007, 2011) & Immigration Regulations (2014) | 2007–2014 | Ensures detainees have access to basic health services. Requires separation based on health and security risk categories. |
| Refugee Amendment Act (2017) & Regulations (2019) | 2019 | Narrows asylum seeker protections, including limits on healthcare, education, and employment rights. |
| National Integrated Sexual and Reproductive Health and Rights Policy | 2019 | Recognizes migrants and asylum seekers as a priority group. Promotes culturally competent, migrant-friendly services and continuity of care. |
| Border Management Act | 2020 | Increases securitisation; restricts mobility and access to services for non-citizens. |
| National Strategic Plan for the Prevention and Control of Non-Communicable Diseases (2020–2025) | 2020 | Outlines goals for achieving SDGs but makes no specific provision for non-citizens. |
| Labour Migration Policy (Draft) | 2022 | Proposes pathways to Universal Health Coverage (UHC) and SDG targets, with a focus on migrant labour inclusion. |
| National Health Insurance (NHI) Act | 2023 | Restricts healthcare access for non-citizens to emergency and notifiable conditions only. |
| White Paper on Citizenship, Immigration, and Refugee Protection (Draft) | 2024 | Proposes stricter immigration criteria. Reviews South Africa’s international refugee commitments to align with national capacity. |
