# Supplementary material for: “Closing the gap in the wrong direction” migration, health policy, and the exclusion of asylum seekers, refugees and undocumented migrants from healthcare access in South Africa
Source: BMC Public Health. 2025 Nov 10;25:3877. doi: 10.1186/s12889-025-24751-4 (PMC12604325; doi:10.1186/s12889-025-24751-4)
Supplement: Supplementary file 2 — Supplementary Material 2. [file 12889_2025_24751_MOESM2_ESM.docx]

INTERVIEW GUIDE FOR KEY INFORMANTS

## Protracted Displacement, Conflict and Gendered Violence

A project exploring the relationship between displacement, gendered violence, and mental ill-health for IDPs, refugees, and asylum seekers who face multiple barriers to accessing healthcare.

RESEARCHER: **Dr Rebecca Walker**

**Telephone:** +27 83 2977911

**Email:** [**bexjwalker@gmail.com**](mailto:bexjwalker@gmail.com)

**TOPICS TO BE COVERED:**

- Health care provision generally and mental health care provision specifically for displaced populations in SA (Congolese and Somali)
- SA health system - policy and practice
- Understanding of migration within the health system
- Relationship between mental health and physical health, both personally and through experience working in a SA context
- Mental health conditions (related to gender, nationality, age, type of migrant, documentation status, current situations etc)
- Gendered experiences of health access and challenges

## PART 1: Background and Professional Role

1. **Could you tell us about your background and current role?**
   a. Place and nature of work
   b. Current and previous responsibilities
   c. Involvement in healthcare or migrant/refugee-related services
2. **What is your understanding of healthcare provision in South Africa, especially in relation to displaced populations?**
   a. Differences between public vs private healthcare
   b. Experiences with nationals vs non-nationals
   c. Awareness of relevant laws, policies, and guidelines (e.g. National Health Act, Refugees Act, NHI)
   d. Observed changes in law, policy, capacity, or attitudes over time
3. **Are you familiar with the UNHCR urban refugee policy and its application in South Africa?**
   a. Is it useful in your work?
   b. What are the practical challenges in implementation?

## PART 2: Health System Access and Policy Implementation

1. **Have you observed any shifts in access to healthcare for refugees, asylum seekers, and undocumented migrants over time?**
   a. Effects of policy reforms e.g the NHI
   b. Impact of immigration law changes
   c. Trends in service access, entitlement perceptions, and patient numbers
2. **What are the most common systemic or practical barriers migrants face in accessing healthcare (including mental health)?**
   a. Documentation, fees, or discrimination
   b. Inconsistent interpretation of policies at the clinic level
3. **Are current laws and policies clear and supportive in enabling equitable access to healthcare for displaced groups?**
   a. Are front-line workers trained in or guided by these frameworks?
   b. What gaps exist in implementation?

## PART 3: Mental Health and Displacement

1. **What mental health issues are most common among displaced populations (especially Congolese and Somali migrants)?**
   a. How do these vary by:
   - Gender
   - Age
   - Nationality or ethnicity
   - Religious group
   - Documentation status
   - Type of migrant (e.g. asylum seeker vs refugee vs economic migrant)
2. **Have you noticed trends or changes in mental health concerns over time or across contexts?**
   a. Differences between urban and rural areas
   b. Role of state, NGO, or community support
   c. Influence of national, regional. global migration politics
3. **How are mental health diagnoses made in practice for these groups?**
   a. Use of DSM or other clinical tools
   b. Recognition of cultural idioms of distress
   c. Tensions between Western diagnosis and lived experience
4. **How useful is the concept of ‘daily stressors’ in explaining mental health challenges for refugees and asylum seekers?**
   a. Examples: fear of deportation, poverty, housing instability, etc.
   b. Links to physical health or chronic conditions

## PART 4: Gender, SGBV, and Mental Health

1. **From your experience, how does gender shape access to mental health services among migrants?**
   a. Do women, transgender, or non-binary people face unique or compounded barriers?
   b. Are services gender-responsive or inclusive?
2. **What is the relationship between sexual and gender-based violence (SGBV) and mental health among displaced populations?**
   a. Is SGBV a widespread issue in the populations you work with?
   b. Are current services and systems adequately equipped to respond?
   c. How can this be improved?

## PART 5: Rights, Gaps, and Future Reforms

1. **What mental health services or needs are most neglected for migrants and refugees?**
   a. Why are these neglected—resources, stigma, lack of training, legal ambiguity?
   b. How do fear, disclosure risks, or mistrust of the system factor in?
2. **If the health system were redesigned to adequately support the health of displaced populations, what would it look like?**
   a. What policies, models, or practices would you recommend?
   b. How could it be made more sensitive to gender, age, nationality, and documentation status?

## PART 6: Final Reflections

1. **Is there anything else you’d like to add, or any questions you think we should have asked?**

**Thank you for your time.**

For any further information on the ethical clearance please contact: [**shaun.schoeman@wits.ac.za**](mailto:shaun.schoeman@wits.ac.za).

Contact details of researchers:

- Rebecca Walker: 0791194863. [**Bexjwalker@gmail.com**](mailto:Bexjwalker@gmail.com)
- Prof Jo Vearey: 0723927034. [**Jovearey@gmail.com**](mailto:Jovearey@gmail.com)

African Centre for Migration & Society (ACMS), Wits University, 1 Jan Smuts Avenue, Braamfontein, Johannesburg
